# Culling Decisions and Dairy Cattle Welfare During Transport to Slaughter in the United States

**DOI:** 10.3389/fvets.2018.00343

**Published:** 2019-01-18

**Authors:** Lily N. Edwards-Callaway, Jennifer Walker, Cassandra B. Tucker

**Affiliations:** ^1^Department of Animal Science, Colorado State University, Fort Collins, CO, United States; ^2^Quality and Food Safety Danone North America, White Plains, NY, United States; ^3^Center for Animal Welfare, Department of Animal Science, University of California, Davis, Davis, CA, United States

**Keywords:** slaugher, cull dairy cow, transport, animal welfare, supply chain

## Abstract

Nearly a third of dairy cows are removed from herds annually in the United States. Our objective is to describe what is known about the process of sending a dairy cow to slaughter in the United States including our perspectives about her fitness for transport, her condition upon arrival at the slaughter plant and the decisions to transport her in the first place. This process begins when the decision is made by the farmer to remove a cow from the herd. Once a cow leaves the farm, she makes her way either directly to slaughter or goes through one or more livestock auctions or markets along the way. Cull cows can travel considerable distance to slaughter and may face a number of welfare challenges during this process. These stressors are exacerbated if the cows are compromised and not fit for transport. While all major industry stakeholders have recommendations or guidelines about fitness for transport, none are enforced rules or regulations. There is little financial disincentive for farmers to stop shipping compromised dairy cows, and, in some cases, slaughter plants are willing to take the risk on purchasing cows in this condition as those that survive the journey often generate a good margin of return. As a result, the decision to ship compromised cull cows is too common, as indicated by data about cow condition both at the farm and the slaughter plant. Compromised culled dairy cattle continue to arrive at slaughter plants and leadership within the industry is needed to tackle this welfare challenge.

## Introduction

In the United States, 28% of dairy cows are removed from dairy herds each year (1). The majority of these animals are culled and are often slaughtered in specialized plants. However, because of the size of the country, this often means that animals are transported long distances. This process is largely unregulated other than cattle cannot be transported for more than 28 continuous hours without at least 5 h of rest (2). Although there are datasets quantifying transport distance for culled dairy cows for certain segments of the trip (i.e., from the farm to the livestock auction/market or from the last pick up point to the slaughter plant), there is a shortage of information about the duration and distance of the entire journey from the place of origin. This is likely, due in part, because cows change owners through this process and each part of the supply chain tracks cow movements in their own way. Our objective is to describe what is known about the process of sending a dairy cow to slaughter, fitness for transport, her condition upon arrival at the plant and how decisions to cull are made in the United States. We write from our perspective as either an academic (Edwards-Callaway, Tucker), as individuals employed by corporations involved in the supply chain (Edwards-Callaway, Walker) and, as an individual that has been involved in these management decisions as a veterinary practitioner (Walker). From these perspectives, we discuss what holds this system in place and opportunities to improve cull cow welfare.

## Cull Cow Transport to Slaughter

Currently, in the United States cows culled from dairies arrive at the slaughter plants through various routes. The majority of dairy operations sold at least some of their culled cows through a livestock market or auction, according to the National Animal Health Monitoring System (NAHMS) of the dairy industry (3) and this approach, used by 92% of operations, accounts for 58% of the culled cow population represented in this survey. Only 37% of operations sent cows direct to slaughter and this varied by herd size and region (3). Selling direct to slaughter can minimize cow transport time as well as total time to slaughter. For example, of those cows that travel direct to slaughter, 50% of animals travel < 80 km, 38% travel 80–400 km and 11% travel more than 400 km (3). In contrast, cows that travel to a livestock market from the dairy farm, 78% travel < 80 km and 22% went 80–400 km on this first leg of their journey. Once sold at the livestock market or auction, most animals will be transported to a slaughter plant. Although the NAHMS data represents 80% of cows nationwide (1), states in the Southeast and parts of the West were not sampled. These regions include states with moderate dairy cow populations and sometimes limited access to large specialized cull cow slaughter plants; the distances described could in fact be longer for some animals depending on their location within the United States.

At each point of sale and during each leg of the route, dairy cattle can be exposed to stressors such as comingling with unfamiliar animals, feed and water deprivation, engorged udders (which can affect mobility and comfort), handling by various people and through multiple facilities, and various transport and environmental conditions [reviewed by (4)]. They also stand for much of the journey. The most recent National Beef Quality Audit (NBQA) provided transport time data on a subset (*n* = 154 loads) of cull dairy and beef cows and bulls arriving at slaughter plants nationwide, indicating that, on average, these cattle were in transit for 6.7 h, with a few loads in transit for over 24 h (5); it should be noted that this in transit duration is from their last stop prior to the slaughter plant and does not necessarily represent the entire duration of travel. Dairy cows rarely voluntarily spend this much time standing (6) and opportunities to rest in trucks are limited to impossible. Cows deprived of both the opportunity to lie down and feed for 3 h will prioritize rest over food when both options are provided again (7) and they will push, on average, 40% of their body weight in order to access a high quality lying area (8). In addition to all of the other stressors associated with transport described above, the limited opportunities to rest are often likely underestimated, as even relatively short trips are likely to affect this aspect of their welfare.

**Figure 1:**
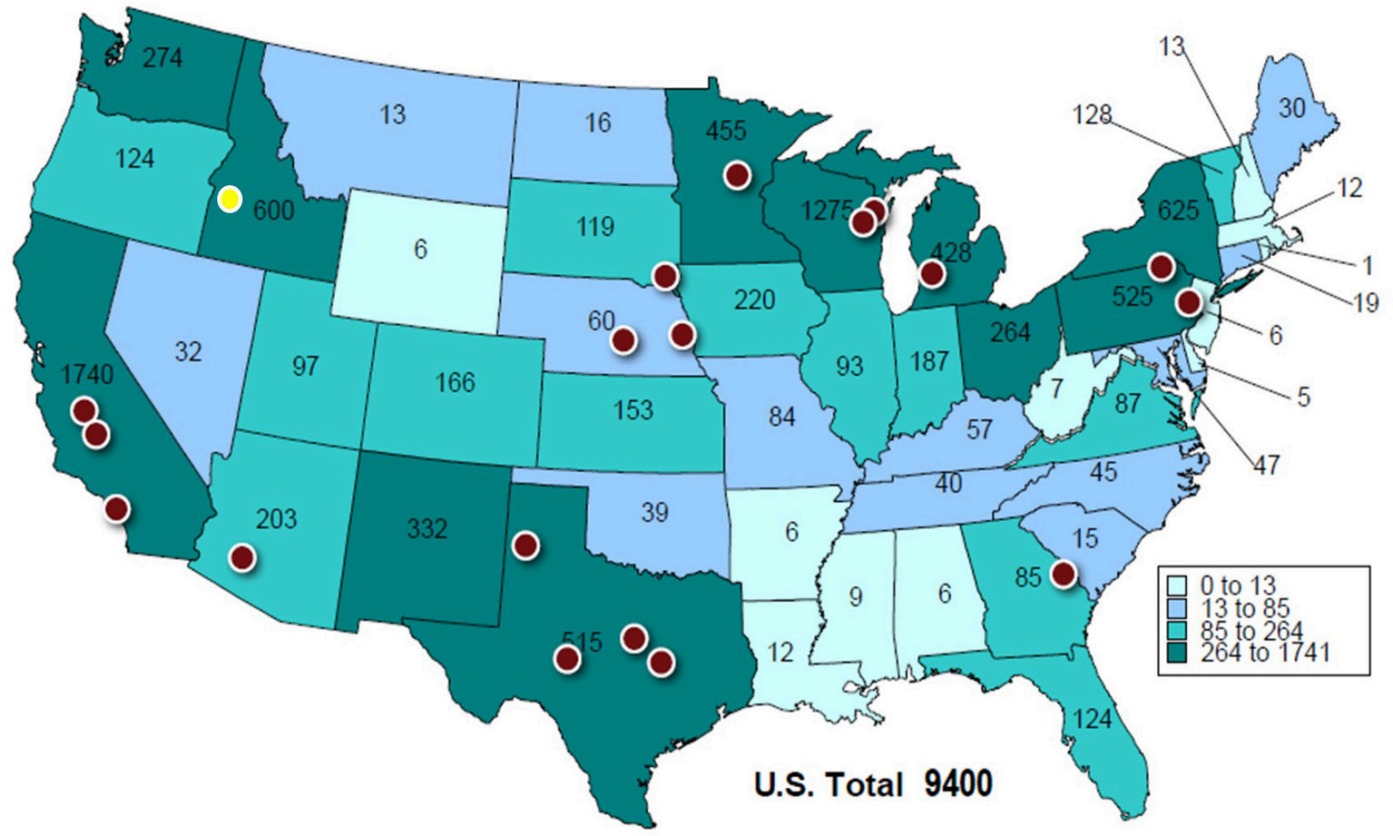
Dairy populations, as indicated by number of cows (1,000 head) calved as of January 1, 2018 (Alaska and Hawaii are not represented in the figure; Alaska = 0 and Hawaii = 2), and locations of 19 specialized slaughter plants that accept cull dairy cows across the United States [(9), adapted by National Cattlemen's Beef Association with permission from the Livestock Marketing Information Center for the use of the map]. The red circles represent slaughter plants that were sampled in the 2016 National Beef Quality Audit. The yellow circle represents a newly-opened slaughter plant (2017). These plants slaughter the vast majority of cull dairy cows in the nation.

Currently in the United States, there is a scarcity of industry data quantifying the *entire* journey a cull dairy cow takes to slaughter. Figure 1 provides initial consideration for the potential journey a cow must take once she is culled. In many of the highly populated milk cow states, there are large commercial slaughter plants, per this map. However, in certain areas, for example in the Pacific Northwest, there are a considerable number of cows and, until recently (2017; yellow circle in Figure 1) there was not a specialized slaughter plant in the region. There likely are smaller local plants within the states that may serve as a final destination, but it is also likely that many animals in these regions travel to the commercial plants in Figure 1. The NAHMS data indicates that 30% of dairy operations send cull cows across state lines when shipping directly to slaughter (3). Additionally, it should be noted that the presence of a large commercial slaughter plant nearby does not preclude a cull cow from being transported a farther distance to a specific slaughter plant based on the buyer's needs for animal type and numbers at certain locations.

To summarize, a large proportion of cull dairy cattle likely have a journey of significant time, distance and challenging conditions as they move through the final stages of the supply chain to slaughter. Transport to slaughter is part of the dairy cow production cycle, and her welfare must be considered. Although this process can be strenuous for any cull cow, it is our perspective that this is an unfair demand for the portion of cows that are compromised and not fit for transport.

## Fitness for Transport

Although the ownership and custody of these cull cows may change during the marketing process, all of the stakeholders are responsible for minimizing stress and pain of these animals. One of the major challenges with cull dairy cattle transport is managing cull cow condition and ultimately fitness for transport. Although the segments of the cattle industry share similar considerations for transport (e.g., lameness, udder condition, wounds, etc.), there is no consensus about a definition for “fitness for transport,” nor is this process regulated in the United States. The National Dairy Farmers Assuring Responsible Management (FARM) Program, supported by the National Milk Producers Federation and Dairy Management, Inc., provides the dairy industry with guidelines and requirements for dairy cow care. The FARM Animal Care Manual includes discussion of fitness for transport primarily within the considerations for culling and euthanasia information (10). The program cites non-ambulatory status, body condition score, imminent calving, dehydration/exhaustion, stage of lactation, injuries, and some other disease-related characteristics as conditions related to decisions for and precluding cows from transport, but there is no verification for adherence to these guidelines required to be a certified participant. The parallel program that oversees animal care at the slaughter plants is the North American Meat Institute Animal Care and Handling Guideline and Audit Tool (11), used by the vast majority of the slaughter industry as the “gold standard” for animal handling. The audit includes a few questions regarding cow condition upon arrival to the slaughter plant, i.e., non-ambulatory cattle, emaciation and udder condition, but none of these outcomes are considered in the final audit score that determines if the plant will meet requirements set by customers. The Livestock Marketing Association (LMA) provides guidance within their employee animal handling training manual on managing injured, disabled, or non-ambulatory animals and recommend that markets “make every effort” to refuse injured or disabled animals (12), but this is not mandated and varies among individual livestock markets. The National Cattlemen's Beef Association (NCBA) Beef Quality Assurance Program has recently launched a new cattle transporter training program which includes a segment on fitness for transport providing both considerations for transport (e.g., mobility, body condition, and health) and the driver's role in making fitness for transport decisions (13). The training includes a segment indicating the importance of these decisions and indicates that the driver has a key role in making the final determination to ship an animal. Although the importance of fitness for transport is addressed in all of these industry programs, the information is a guideline or recommendation, not a requirement or rule.

## Current Condition of Cull Cows Arriving at Slaughter

The NCBA has been funding and coordinating national audits aimed to quantify the condition of cattle arriving at slaughter plants, in addition to other objectives, since the early nineties. The 2016 NBQA indicated that 9% of dairy cows were extremely thin, 43% had a defect (such as a swollen joint or foot abnormality) and 23% were mobility-challenged (5). In 2014, a large survey was conducted to benchmark the prevalence of several cattle health problems (i.e., severe lameness, body condition score, udder condition, prolapse, cancer eye, malaise, wounds, active parturition, nervous system disorder, non-ambulatory) of cull cows arriving at slaughter plants that supplied to a specific multinational company (14). Within the population of United States dairy cattle sampled (*n* = 8,601), 9% of the cattle had one or more identified welfare problems. It should be noted that this survey focused only on the extreme cases of lameness, emaciation and injury, which may explain why the overall prevalence of welfare problems was lower than determined in the NBQA. Many of the problems identified in the aforementioned studies are ones that likely did not occur during the transport process, but were evident at the time the animals were shipped from the dairy.

## Culling Decisions on Dairies

Culling is typically considered in 2 separate categories: voluntary, cows culled purely based on productivity (milk production) and involuntary, cows culled as the result of an underlying health issue including but not limited to infertility, lameness, mastitis or injury [e.g., (15)]. In practice, as evidenced by recommendations offered in farmer facing trade magazines (https://www.dairyherd.com/article/when-cull-or-not) and in peer reviewed publications [e.g., (16)], voluntary culling decisions in the US are primarily influenced by milk price, cull cow value at market or slaughter, reproductive status, replacement costs and housing capacity. Based on our experience, these decisions usually take place on a weekly basis or, at best, twice a week because livestock markets receive cull cows on designated days each week. For example, once the value of a cow's daily milk production, which is dependent on a fluctuating milk price, is no longer able to cover feed cost, she would make a farm's list of potential voluntary culls (17). This break-even in production may be as little as 7.3 kg per cow per day when milk price is high and feed costs are low, or as high as 27 kg when milk price is low and feed costs are high, the range often being 18–27 kg of milk per day (for example, https://afs.ca.uky.edu/dairy/extension/culling-decisions-market-conditions). Using this information, many farms generate a weekly list of cows that are below the break-even point for milk production. Farms often then consider the cows' pregnancy status [e.g., (16)] and, in some cases, may also visually inspect the animals on the list. Our professional experience is that once milk production has slipped below profitable levels, then the health and welfare of the cow is given consideration in this type of decision.

Involuntary culling because of a health concern in many instances may be best described as emergency culling. The welfare implications of these late decisions are alarming, as a disturbingly high proportion of compromised cows are sold. Thirty-six percent of cows with cancer eye are sold, 20% with displaced abomasum, 19% of those that had been down for at least 24 h and 15% of cows that are lame (3). The suffering associated with sending cows with these health problems to slaughter are considerable. In the case of a displaced abomasum, for example, this may mean a cow is sent to a livestock market and then to a slaughter plant with part of her stomach partially or completely twisted, and unable/willing to drink or eat for the duration of the journey. Although some farmers and veterinarians may have a clear understanding of what the marketing and transport process will involve in regards to the stressors for the cow, the willingness to put compromised cows on a truck could also be due to a lack of awareness or control over the process and her fate.

## Why?—Market Dis/Incentives

Compromised dairy cattle, unfit for transport, arrive at the slaughter plants because there is no significant disincentive for selling and/or purchasing them. While there are several reasons to not ship a compromised cow, the primary and most critical reason being her welfare, unfortunately, there are also financial incentives for shipping these cows that vary by location within the supply chain. The decision to ship a cull cow begins at the dairy farm. If the farmer believes the cow has a chance at surviving the journey to sale or slaughter, the benefit of shipping her is the profit of the sale price. Additionally, when compromised cows are shipped, euthanasia is not performed on-farm, resulting in savings associated with this process and disposal expenses. At the livestock market, the ownership of the cow changes from the farmer (consignor/seller) to the buyer. If euthanasia is required while at the livestock market facility, the decision is often left to individual owners. Although the livestock market may face the potential cost of additional handling and carcass disposal if a cow dies on the premises, there is a larger risk of potentially losing a consignor's future business by turning away high-risk animals. The decision to ship a cow from the livestock market/auction to the slaughter plant is made by the buyer, who could be an employee of the slaughter company or an independent dealer. From the perspective of the buyer, the benefit of buying this type of cow, assuming she makes it to slaughter, is the margin made on processing a lean cow. However, there are also significant risks. High-risk cows may not survive the trip to slaughter or they may not pass regulatory inspections at the slaughter plant and, in these instances, the entire value of the cow is lost, both the purchase price and freight cost. Handling compromised cattle is difficult and often closely scrutinized by in-plant regulatory bodies. Additionally, the image of a compromised dairy cow trying to move through a slaughter plant is often not in alignment with retailer/customer expectations and visions for their food supply. Companies who purchase and slaughter cull dairy cows try to buy cows that are near to their plants with minimal stops in-between, as often these animals present a higher risk for death loss and carcass shrink (a negative carcass outcome) due to their condition as the transport duration increases. We are not suggesting that finances always dictate fitness for transport decisions, but want to acknowledge that these financial components often play a significant role in decision-making. It is clear that the current system does not satisfactorily discourage the shipment of dairy cows that are unfit for transport.

## Conclusion

A key frustration for many working in this area is that compromised cows continue to be marketed and transported and that this has been going on for some time. For example, in 1994 when the NBQAs were initiated, Dr. Gary Smith made a timeless statement regarding cull cow marketing: “It all boils down to timely marketing and management. When a cow's productivity goes downhill, get her to market. When you know her teeth are gone, get her to market. When she's a little bit lame, get her to market” (18). This was over 2 decades ago and likely not the first time that timely marketing of animals was discussed, and certainly not the last. Regulation of this problem and fining non-compliant producers, as is done in Europe, is highly unlikely in the United States. However, change through the supply chain is possible with leadership and understanding of the challenge. What holds the current system in place, preventing significant change and improvement when so many within the supply chain identify cull cow condition as an important welfare concern? In part, there could be a lack of understanding by some within the supply chain of the entire journey a cull cow must make once she leaves the farm, as these data are not routinely collected. Additionally, as illustrated, stakeholders within the supply chain make decisions focused on production and finances rather than cow welfare. Finally, there seems to be a shared inertia within the supply chain to make a significant change in the system. Although decisions begin on the dairy, all stakeholders in the livestock market, transport, and slaughter process have the responsibility and the ability to protect the welfare of dairy cattle. Leadership is needed to address the challenges associated with culling decisions and dairy cattle welfare during transport to slaughter in the United States.

## Author Contributions

All authors listed have made a substantial, direct and intellectual contribution to the work, and approved it for publication.

### Conflict of Interest Statement

The authors declare that the research was conducted in the absence of any commercial or financial relationships that could be construed as a potential conflict of interest.
